# Supplementary material for: Acceptance and compliance with micronutrient powder (MNP) among children aged 6–23 months in northern Nigeria
Source: PLOS Glob Public Health. 2022 Oct 17;2(10):e0000961. doi: 10.1371/journal.pgph.0000961 (PMC10022258; doi:10.1371/journal.pgph.0000961)
Supplement: S1 Dataset — (ZIP) [file pgph.0000961.s008.zip › S8_Dataset/Direct Observations.zip!/Direct Observations/230605_A_GA_AD_1_DO.pdf]

1.1.1 Instrument F –Direct Observation Form

<<Instrument F Direct Observation Form>>

PI Name: PragyaMathema,Stephen Kodish
Study Title: Formative research to develop an infant and young child feeding (IYCF) intervention with micronutrient powder (MNP) for children aged 6 – 23 months
PI Version/Date: Version1.0/January 24, 2015

Date: _June 23, 2015_____________Location: _ Ganwoki community, GANYE, LGA_

Data Collector: Aisha Aliyu Dahiru____Participant ID: _Round hut with mango tree in front

Main goals of this direct observation are to answer the following questions:

1. In this household, what would be the best way(s) to give nutritional supplements to young children? Why?

2. In this household, describe the food sharing that you observe throughout the meal. Are there any predictable patterns?

3. In this household, who is favored and how is this favoritism expressed during meal preparation and eating?

(TEAR OFF AT PERFORATED DOTTED LINE BELOW)

PARTICIPANT ID: __

Household Composition Table (Fill out as you do data collection)

INITIALS	SEX (M/F)	AGE (estimated)	Relationship to household	Description
W	F	26YRS	Mother	Wearing pink wrapper agnd cream shirt.
C	M	13 MONTHS	Baby boy (son)	Wearing white sweater
H	M	34 YEARS	Husband	Wearin brown kaftan and white boots
OC	F	11 YEARS	Daughter	Wearing black shirt and green wrapper
N1	F	27YEARS	Mother friend	Wearing orange wrapper with brown head gear
N2	M	30 YEARS	Husband's sister	Wearing orange shirt and black trouser gear
G	F	15YRS	Wife sister	Wearing blue dress

B1	M	6 years	Daughter	Wearing brown kaftan and black cup
B2	M	4 years	Son	Wearing blue sweat shirt
N3	M	21 years	Husband's nephew	Wearing ash cloth
N4	F	30 years	Neighbour	Wearing green shirt and brown wrapper

PARTICIPANT ID: __________

TIME	ACTOR INITIALS	RECIPIENT INITIALS	WHAT HAPPENED	FOOD	Reactivity
8.00	M	C	Carrying baby on laps		
8:05	M	C	Carrying baby on laps		
8:05	OC		Sweeping the verandah		
8:10	OC		Sweeping the verandah		
8:15	M	C	Breastfeeding the baby		
8:20	M	C	Breastfeeding the baby		
8:25	H		Comes into the house holding a radio		
8:30	C		Playing on the mother's lap and pointing		
8:35	M	C	Breastfeeding the baby		
8:35	OC		Mopping the verandah		
8:35	H		Comes out of the room holding radio and hoe		
8:40	H	M	Chatting with mother		

TIME	ACTOR INITIALS	RECIPIENT INITIALS	WHAT HAPPENED	FOOD	Reactivity
08:45	H	M	Chatting with mother		
08:50	H		Goes out talking about the heavy rainfall		
08:55	B1		Washing his feet		
08:55	B2		Playing with baby under the shade		
09:00	C		Running around		
09:00	H		Comes in and changes clothes		
09:05	H		Picks up cutlass, hoe and his radio and goes out		
09:05	H	M	Telling mother to ensure she gives the child MNP		
09:10	M	OC	Chatting with daughter		
09:15	M		Bring out flask and cup from the room		
09:15	M		Serve a portion of kunu from flask into a cup	Kunu gyada	
09:20	M		Adds MNP into the Kunu	Kunu gyada	
09:20	M		Mixing the MNP ensuring it blends into the Kunu	Kunu gyada	
09:25	M	C	Feeding the baby kunu with spoon	Kunu gyada	
09:26	M	C	Feeding the baby	Kunu gyada	
09:27	M	C	Feeding the baby. The other children were not present	Kunu gyada	
09:28	C		Opening mouth to be fed eager to accept the kunu	Kunu gyada	
09:29	M	C	Feeding the baby	Kunu gyada	
09:30	M	C	Feeding the baby	Kunu gyada	
09:35	M	C	Washing the baby’s hands nd mouth		
09:40	M	C	Carrying the baby on her lap		

TIME	ACTOR INITIALS	RECIPIENT INITIALS	WHAT HAPPENED	FOOD	Reactivity
09:40	M	C	Playing with baby		
09:40	N1	M	Comes in greeting the mother		
09:40	M	N1	Chatting with friend		
09:40	G		Picking groundnut in a large bowl		
09:45	M	N1	Chatting with friends		
09:45	OC		Getting ready for school		
09:50	M	C	Breastfeeding baby	Breast milk	
09:55	M	C	Breastfeeding baby	Breast milk	
09:55	C		Playing on mother's lap		
10:00	C		Playing on mother's lap		
10:05	G		Picking utensils around the house into the kitchen		
10:05	B2	C	Playing with the baby		
10:10	C		Crying		
10:10	M	C	Pick up the baby and consoling baby on her lap		
10:15	M	C	Breastfeeding baby	Breast milk	
10:20	N2	M	Comes in greeting mother		
10:20	M	N2	Chatting with husband's brother		
10:25	M	N2	Chatting with husband's brother		
10:30	N2		Exiting the house		
10:30	M	C	Washing the baby		
10:30	C		Baby crying		
10:35	M	C	Consoling baby		
10:40	N3	M	Comes into house greeting mother		
10:40	N3	M	Chatting with mother		
10:45	M	N3	Chatting with husband's nephew		
10:50	M	C	Breastfeeding baby	Breast milk	
10:55	M	C	Laying with baby on her lap		
10:55	M	C	Breastfeeding baby	Breast milk	
10:56	M	C	Breastfeeding baby	Breast milk	
10:57	M	C	Breastfeeding baby	Breast	

TIME	ACTOR INITIALS	RECIPIENT INITIALS	WHAT HAPPENED	FOOD	Reactivity
				milk	
10:58	M	C	Breastfeeding baby	Breast milk	
10:59	M	C	Breastfeeding baby	Breast milk	
11:00	M	C	Carrying baby on her back		
11:05	M	C	Carrying baby on her back		
11:10	M	C	Carrying baby on her back		
11:10	M		Washing utensils that was left under the rain		
11:15	M		Goes into the room and brings clothes out		
11:15	M		Soaking clothes and utensil in a large bowl		
11:20	M		Carrying baby on her back		
11:20	M		Picking utensils around the house		
11:25	M	C	Carrying baby on her back		
11:25	B2		Sitting under the shade playing		
11:30	M		Bringing out fruit from the kitchen and hangs on veranda	Local grapes	
11:35	C		Humming on mother's back		
11:35	M		Moving bags from one corner of the room to the other		
11:40	N4		Comes into the house holding a bucket		
11:40	M	N4	Chatting with neighbor		
11:45	M	N4	Chatting with neighbor		
11:45	M		Carrying baby on her back		
11:50	M	N4	handing neighbor utensils used for drawing water from the well		
11:50	N4		Goes out chatting with mother		
11:50	M	N4	Going out with neighbor		
11:50	M	C	Carrying baby on her back		
11:55	M	N4	Chatting with neighbor near the well in front of house		
11:55	M	N4	Chatting with neighbor		

TIME	ACTOR INITIALS	RECIPIENT INITIALS	WHAT HAPPENED	FOOD	Reactivity
12:00	M	N4	Chatting with neighbor		
12:05	M	C	Carrying the baby on her back walking back into the house		
12:10	M	G	Chatting with sister		
12:15	M	G	Chatting with sister		
12:15	M	C	Brings baby down from back		
12:15	M		Spreads wrapper on the veranda and lays down		
12:15	M	C	Lie baby down beside her		
12:20	M	C	Breastfeeding baby	Breast milk	
12:25	M	C	Breast feeding baby	Breast milk	
12:25	M	G	Chatting with sister		
12:30	M	G	Chatting sister		
12:35	M	C	Playing with the baby		
12:40	M	C	Playing with baby		
12:45	M	G	Chatting with sister		
12:45	M	C	Playing with baby		
12:50	C		Running around the veranda laughing		
12:55	C		Following mother into the room		
12:55	M	B2	Mother serving kunu to the older son	Kunun gyada	
12:55	B2		Sitting and drinking kunu	Kunu gyada	
12:55	C		Sitting beside brother playing with cap		
01:00	B2	C	Offering kunu to the baby		
01:00	C		Refusing to take kunu from brother		

TIME	ACTOR INITIALS	RECIPIENT INITIALS	WHAT HAPPENED	FOOD	Reactivity
01:00	M		Folding clothes on the bed		
01:00	C	C	Following mother around the house (from one room to the other)		
01:05	M	C	Picking clothes from one room to the other		
01:10	M	C	Carrying baby		
01:15	M	C	Brings down baby in the room		
01:15	M		Picking clothes from room to veranda		
01:20	M	C	Changing baby's clothes		
01:20	M		Folding clothes		
01:20	B2		Finished drinking kunu and take cup into the kitchen		
01:20	B2		Joining mother and baby in the room		
01:25	B2	C	Playing with baby in the room		
01:30	B2	C	Playing with baby in the room		
01:35	B2	C	Playing with baby in the room		
01:40	B2	C	Playing with baby in the room		
01:40	G		Sitting on the veranda		
01:40	M		Folding clothes in the room		
01:45	B2	C	Playing with baby in the room		
01:45	M		Bringing out more clothes to the veranda		
01:50	C		Playing with brother and giggling		
01:50	B2	C	Playing with baby		
01:55	C		Giggling and peeping outside		R
02:00	B2	C	Playing with baby at the veranda		R
02:00	M	G	Chatting with sister		
02:05	C		Running around and screaming loudly		R
2:10	B2	C	Playing with baby in the room		
02:10	M		Sweeping the room		
02:10	B2	C	Playing at the veranda with baby		
02:15	M		Sweeping the room		
02:15	G	B2,C	Playing with the children		
02:20	M		Sweeping the veranda		
02:20	G	B2, C	Playing with the children		
02:25	M		Sweeping the surrounding		

TIME	ACTOR INITIALS	RECIPIENT INITIALS	WHAT HAPPENED	FOOD	Reactivity
02:25	M	G	Chatting with sister		
02:30	M		Sweeping the surrounding		
02:35	B2	C	Playing around with the baby at the veranda		
02:35	M		Sweeping the surrounding		
02:40	C		Running around, screaming and laughing		
02:45	C		Running around, screaming and laughing		
02:45	B2	C	Running around with the baby screaming		R
02:45	M		Sweeping the surrounding		
02:50	B2	C	Playing in the room with the baby		
02:50	M		Sweeping the surrounding		
02:50	M		Washing hands after sweeping		
02:50	G		Picking beans in a silver utensils		
02:50	M		Resting at the veranda with baby and older son		
02:50	B2	C	Playing with baby		
02:55	M	C	Carrying baby on her lap		
02:55	M	C	Breastfeeding baby		
03:00	B2	C	Playing with baby near the mother		
03:05	B2	C	Playing with baby in the room		
03:05	M	G	Chatting with sister		
03:10	B2	C	Playing with baby in the room		
03:15	M		Loosening plaited hair		
03:20	C	M	Playing with mother’s hair		
03:25	C		Playing with brother		
03:30	C		Standing beside mother quietly		
03:35	C	M	Playing with mother’s hair		
03:35	M		Loosening plaited hair		
03:35	M	G	Chatting with sister		
03:40	G	C	Carrying baby on lap		
03:40	G	C	Playing with baby. Lifting baby up and down.		
03:45	M	C	Carrying baby on her laps		
03:50	C	B2	Hit brother playfully with comb		
03:50	B2	C	Play and running around with baby		
03:55	M		Combing hair		
04:00	C	B2	Hit brother playfully with comb		

TIME	ACTOR INITIALS	RECIPIENT INITIALS	WHAT HAPPENED	FOOD	Reactivity
04:05	G	C	Carrying baby on her lap		
04:10	B2	C	Playing with baby		R
04:15	B2	C	Playing with baby		R
04:15	M	G	Chatting with sister		
04:20	B2	C	Playing hide and seek with baby		
04:25	B2	C	Playing and running around with baby		R
04:30	M	C	Giving baby water to drink in a cup	water	
04:35	G	B2,C	Playing with children in the room		
04:40	G	B2,C	Playing with children in the room		
04:45	B2		Running out of the house giggling		
04:50	G	C	Playing with baby in the room		
04:55	M		Loosening hair		
04:55	M	G	Chatting with sister		
05:00	G	M	Shuffling through pictures and chatting with sister		
05:00	C		Playing with some pictures while going up and down in the veranda		
05:05	C	M	Pointing out picture to mother and screaming		
05:10	M	C	Carrying baby on her lap		
05:10	M	G	Chatting with sister		
05:15	C	M,G	Pointing out picture and screaming to mother and aunt		
05:20	C		Lying down beside mother at the veranda		
05:20	M	G,B2,B1	Chatting with sister and children		
05:25	C		Sleeping beside mother		
05:30	C		Baby is sleeping		
05:35	C		Baby sleeping		
05:40	C		Baby sleeping		
05:45	C		Baby wakes up		

TIME	ACTOR INITIALS	RECIPIENT INITIALS	WHAT HAPPENED	FOOD	Reactivity
05:45	M	C	Carrying baby on her lap		
05:50	M	C	Giving baby kunu in a small cup while sitting on her lap	Kunu gyada	
05:51	M	C	Feeding baby with kunu on her lap	Kunu gyada	
05:52	M	C	Feeding baby with kunu without forcing the baby	Kunu gyada	
05:53	M	C	Feeding baby kunu	Kunu gyada	
05:54	M	C	Feeding baby with kunu	Kunu gyada	
05:55	M	C	Feeding baby with kunu	Kunu gyada	
05:56	M	C	Washing baby's hands and mouths		
05:56	M		Starting fire		
06:01	M		Starting fire		
06:06	M		Placing pot with water on fire	Water	
06:06	M		Covering pot		
06:11	M		Adding guinea corn flour to water on fire to make meal	Guinea corn flour, water	
06:16	M		Meal is ready to be served	Tuwon dawa	
06:21	M		Placing another pot of soup on fire	Miyan kuka	
06:26	M		Soup is ready		
06:31	M		Serving tuwo in a large bowl and soup in a separate bowl	Tuwon dawa, baobab soup	
06:31	G		Placing pot with water on the fire	Water	
06:36	G		Mixing groundnut paste and guinea corn flour in a bowl with water	Guinea corn flour, ground nut paste, water	

TIME	ACTOR INITIALS	RECIPIENT INITIALS	WHAT HAPPENED	FOOD	Reactivity
06:41	G		Adding guinea corn flour and groundnut paste mixture to the boiling water to make kunu		
06:46	G		Kunu is ready to be served	Kunu gyada	
06:51	G		Serving kunu into flask and keep aside	Kunu gyada	
06:51	C		Baby sleeping		
06:56	C		Baby sleeping		

PARTICIPANT ID: __ Round hut with mango tree in front

Primary Questions (Complete after you complete data collection):

From what you have seen observing THIS HOUSEHOLD, please answer the following questions in detail:

Question	Write your detailed response here
1. In this household, what would be the best way(s) to give nutritional supplements such as an MNP to young children to ensure compliance?<br><br>Why?	The baby is fed separately. The mother and sister can be trained on how to use MNP because in the mother's absence the sister is the one taking care of the baby and also administer the MNP.
2. In this household, who is favored during meal preparation or feeding?<br><br>How is this favoritism expressed?	The parents and older children were favored during meal preparation because the meal prepared was solid. Even though a semisolid food was prepared the younger children were also given the semisolid food as well as the baby.
3. How could we promote the MNP supplement for this family that would attract their attention?	MNP can be promoted to this family through the mother and sister.<br>The father is in charge of all decisions in the household so he might serve as an influence in promoting MNP .

Who would they listen to?	The community leader can also serve as an influence because his wife is a friend to the mother of the household and when she visited they were discussing about ways to promote MNP in the community.<br>The radio can also be used to promote the MNP because the man was listening to the radio when he came into the house.
4. In this household, what would be the best ways to promote the MNP but limit how much it would be shared?<br><br>Why?	Ensure the mother understands MNP is meant for her 11 months old baby only and should not be shared with other children because it will only benefit the baby and not the rest.<br><br>Ensure the mother feeds the child the MNP separately and ensure the baby eats the entire portion that is mixed with MNP. The child should be fed before or after the parent and older children have eaten.
5. What other issues and concerns did you see with this family that are important for us to consider with regard to introduction of an MNP?<br><br>Why?	There are no concerns or issues with regard to introduction to MNP in this household.<br>The mother maintains good hygiene and there is adequate food in the household. The baby consumed the portion of kunu that was mixed with MNP with no difficulties.
6. Based on your experience observing this child today, do you believe reactivity negatively impacted your ability to see the food-related behaviors of the child?<br><br>Please explain your answer in detail.	There was no reactivity during food related behavior. The reactivity witnessed was when the child was playing with his mother and older son. The child ate his meal without noticing data collector presence. It is only after eating that he kept playing around near data collector.
